# Causal Effect of Immunocytes, Plasma Metabolites, and Hepatocellular Carcinoma: A Bidirectional Two-Sample Mendelian Randomization Study and Mediation Analysis in East Asian Populations

**DOI:** 10.3390/genes15091183

**Published:** 2024-09-09

**Authors:** Xilong Tang, Jianjin Xue, Jie Zhang, Jiajia Zhou

**Affiliations:** 1 Guangdong Provincial Key Laboratory of Malignant Tumor Epigenetics and Gene Regulation, Sun Yat-Sen Memorial Hospital, Sun Yat-Sen University, Guangzhou 510120, China; 2 Department of Surgery, Sun Yat-Sen Memorial Hospital, Sun Yat-Sen University, Guangzhou 510120, China

**Keywords:** hepatocellular carcinoma, immunocytes, mendelian randomization, mediation analysis, plasma metabolites

## Abstract

**Background**: Hepatocellular carcinoma (HCC) is a primary malignant liver tumor characterized by a low survival rate and high mortality. This study aimed to investigate the causal effect of immune cell phenotypes, plasma metabolites, and HCC in East Asian populations. **Methods**: The summary results for 731 immunocytes, 1400 plasma metabolites, and HCCs were acquired from publicly available genome-wide association studies (GWASs). This study utilized two-sample Mendelian randomization (MR) analysis to establish causal relationships, which was achieved by employing various statistical methods including inverse variance-weighted, simple mode, MR–Egger, weighted median, and weighted mode. Multiple sensitivity analyses were conducted to confirm the reliability of the MR data. Ultimately, mediation analysis was employed to ascertain the path that leads from immunocytes to plasma metabolites. **Results**: Among the 20 immune cells and HCC for East Asians, causal links were found, with one showing an inverse correlation. In addition, 36 metabolites were significantly associated with HCC for East Asians. Through analysis of established causative metabolites, we identified a strong correlation between the glycerophospholipid metabolic pathway and HCC for East Asians. By employing a two-step MR analysis, we identified 11 immunocytes that are causally linked to HCC for East Asians through the mediation of 14 plasma metabolites, with Linolenate [α or γ; (18:3n3 or 6)] levels showing the highest mediation proportion (19.3%). **Conclusions**: Our findings affirm the causal links among immunocytes, plasma metabolites, and HCC in eastern Asia populations by calculating the percentage of the impact that is influenced by plasma metabolites. This study offers innovative perspectives on the early detection, diagnosis, and therapy of HCC.

## 1. Introduction

Hepatocellular carcinoma is one of the most prevalent malignancies within the digestive system [1]. According to 2018 statistics, HCC ranks as the sixth most prevalent form of cancer and the fourth highest contributor to cancer-related deaths on a global scale [2]. The pathogenesis of HCC is intricate, involving multiple risk factors, mainly hepatitis B and C infections, cirrhosis, excessive alcohol intake, obesity, non-alcoholic fatty liver disease, aflatoxin exposure, and diabetes [3]. Chemotherapy, radiation therapy, transplantation of the liver, and surgical excision are the current therapeutic options for HCC [4]. Because of the insidious onset, HCC is often diagnosed at an advanced stage, precluding surgical intervention and resulting in a very poor prognosis [5]. The five-year survival rate for patients with advanced HCC is dishearteningly low at 15% [6].

Immune cells comprise both innate and adaptive immune cells. Innate immune cells include granulocytes, macrophages, and dendritic cells (DCs), whereas adaptive immune cells consist of B cells and T cells [7]. During a condition of equilibrium, these cells remain inactive. However, they swiftly become engaged and react when the body is provoked by infection, inflammation, or other external agents [8]. Among the various immunocyte types, T cells are crucial for pathogen clearance and tumor cell destruction [9]. Research has indicated that elevated levels of CD3 and CD8 T cells, present both within the tumor and at the tumor boundary, are associated with reduced recurrence rates and extended periods of recurrence-free survival [10]. In addition, a dense infiltration of natural killer cells is associated with improved mortality in patients with HCC [11]. Conversely, elevated expression of herpes virus entry mediators in HCC patients has been linked to a more aggressive disease phenotype and poorer overall survival (OS) [12]. While systemic molecular treatments have traditionally been the primary treatment for advanced HCC for over ten years, the introduction of immunotherapies, specifically, immune checkpoint inhibitors, is significantly transforming HCC management [13].

Although previous studies, including those by Chen et al., Zhu et al., and Tao et al., have established a correlation among immune cells, plasma metabolites, and HCC [14,15,16], the exact causality and the specific levels of their mediation have not been completely clarified. MR analysis is a powerful method that utilizes genetic variants as instrumental variables (IVs) to deduce a potential causal relationship [17]. By randomly assigning genetic variations at conception, this approach reduces the impact of distracting variables on causal assessment. Mediation analysis has been employed to assess the effect of exposure factors on outcome factors via mediators [18]. Consequently, we conducted MR studies using publicly accessible GWAS data sets to assess the causality link among immunocytes, plasma metabolites, and HCC and to uncover the routes by which immunocytes influence HCC by means of plasma metabolites.

## 2. Methods

### 2.1. Study Design

We obtained GWAS data sets that included immunocytes, plasma metabolites, and HCC. Then, two-sample MR analyses were used to evaluate the causal relationship among immunocytes, plasma metabolites, and HCC. Finally, a two-step MR analysis was employed to ascertain the mediation impact of plasma metabolites on the association between immunocytes and HCC. This MR study was carried out in accordance with the STROBE-MR protocol [19].

### 2.2. Data Sources

The GWAS Catalog was used to obtain summary data on plasma metabolomics. The research accession codes GCST90199621–GCST90201020 were used, which encompassed 1091 plasma metabolites and 309 metabolite ratios from a total of 8299 European participants [20]. Additionally, the study accession numbers GCST90001391–GCST90002121 offered a comprehensive compilation of GWAS statistics for all immunological traits, including 3757 individuals from Europe and comprising 731 immunocytes [21]. The genetic data for HCC with the GWAS ID bbj-a-158 were acquired from the IEU Open GWAS database [22].

### 2.3. Instrument Variables

In order for causal estimates derived from MR analysis to be considered trustworthy, the following three key assumptions must be satisfied: (1) IVs are correlated with exposures; (2) IVs are not correlated with confounding variables; and (3) IVs do not have a direct relationship with outcomes but affect outcomes through their influence on exposures. More precisely, the IVs included in this study were carefully examined to ensure they met the following criteria: (1) The single nucleotide polymorphism (SNP) that met the genome-wide significance threshold of *p* < 5 × 10*^−^*^5^ was considered as a possible variable connected to all exposure traits [23]. (2) SNPs that were associated with outcome variables were removed from the analysis if their *p*-values were less than 0.05. (3) The clumping method was carried out to mitigate the influence of linkage disequilibrium, specifically when the value of r2 was less than 0.001 and the distance between variants was greater than 10,000 base pairs. (4) The MR pleiotropy residual sum and outlier test were utilized to identify horizontal pleiotropy, and the impact of pleiotropy was mitigated by excluding outliers [24]. (5) According to the “rule of thumb”, the strength of the chosen SNPs was assessed using the F-statistic. SNPs with an F-statistic value less than 10 were removed to prevent any bias caused by weak instruments in the MR analysis [25,26]. Alcohol and cigarette consumption are risk factors for HCC and may influence MR findings as confounding factors [27,28,29]. We searched the LD link database, aiming to find and exclude genetic variants that are directly linked to alcohol and cigarette intake and are often utilized as IVs.

### 2.4. Statistical Analysis

#### 2.4.1. Two-Sample MR

The MR method was employed to assess the causative associations among immunocytes, plasma metabolites, and HCC. Multiple methods including IVW, simple mode, MR–Egger, weighted median, and weighted mode were applied to determine causality for exposures. IVW often offers the highest level of statistical dominance, while the remaining methods serve as supplementary approaches. Based on the MR analysis, it was found that when *p* < 0.05, there was a statistically significant causal association between exposure factors and outcome factors. A Benjamini–Hochberg false discovery rate (FDR) correction was applied in the “validation” and “discovery” analyses in order to perform multiple checks. FDR values less than 0.05 were categorized as “strong evidence” in our studies, while findings falling between FDR values of 0.05 and less than 0.20 were categorized as “suggestive evidence” [30,31].

#### 2.4.2. Reverse MR Analysis

For the purpose of investigating the potential causative impact of HCC on the observed immunocytes (PIVW < 0.05), we conducted a reverse MR analysis.

#### 2.4.3. Analysis of Metabolic Pathways

The analysis of metabolic pathways was conducted on the identified metabolites using MetaboAnalyst 6.0 (https://www.metaboanalyst.ca/), accessed on 1 July 2024 [32]. This work utilized two databases, the Small Molecule Pathway database and the Kyoto Encyclopedia of Genes and Genomes database [33,34].

#### 2.4.4. Mediation Analysis

Our study conducted a two-step MR for mediation analysis to examine if plasma metabolites act as mediators in the pathway from immunocytes to HCC. The mediation effect was calculated using a two-step MR as follows: mediation effect = beta1 × beta2. The overall impact of the immunophenotype on HCC was determined in the earlier two-sample MR analysis. The direct effect was calculated as the difference between the total effect and the mediation effect, and the mediation proportion was determined by applying the following formula: (mediation effect/total effect) × 100%.

#### 2.4.5. Sensitivity Analysis

The MR–Egger and IVW techniques were utilized to conduct heterogeneity testing. The heterogeneity among IVs was evaluated by using Cochran’s Q test, where a *p*-value greater than 0.05 indicated the absence of substantial heterogeneity. In addition, a *p*-value greater than 0.05 indicated the absence of horizontal pleiotropy by employing the MR–Egger regression equation. Eventually, we performed a leave-one-out sensitivity analysis to determine if a single SNP had a significant impact on the causal estimation. Our MR analyses were conducted using the “TwoSampleMR” package in R software (version 4.3.2) [35].

## 3. Results

**Figure 1:**
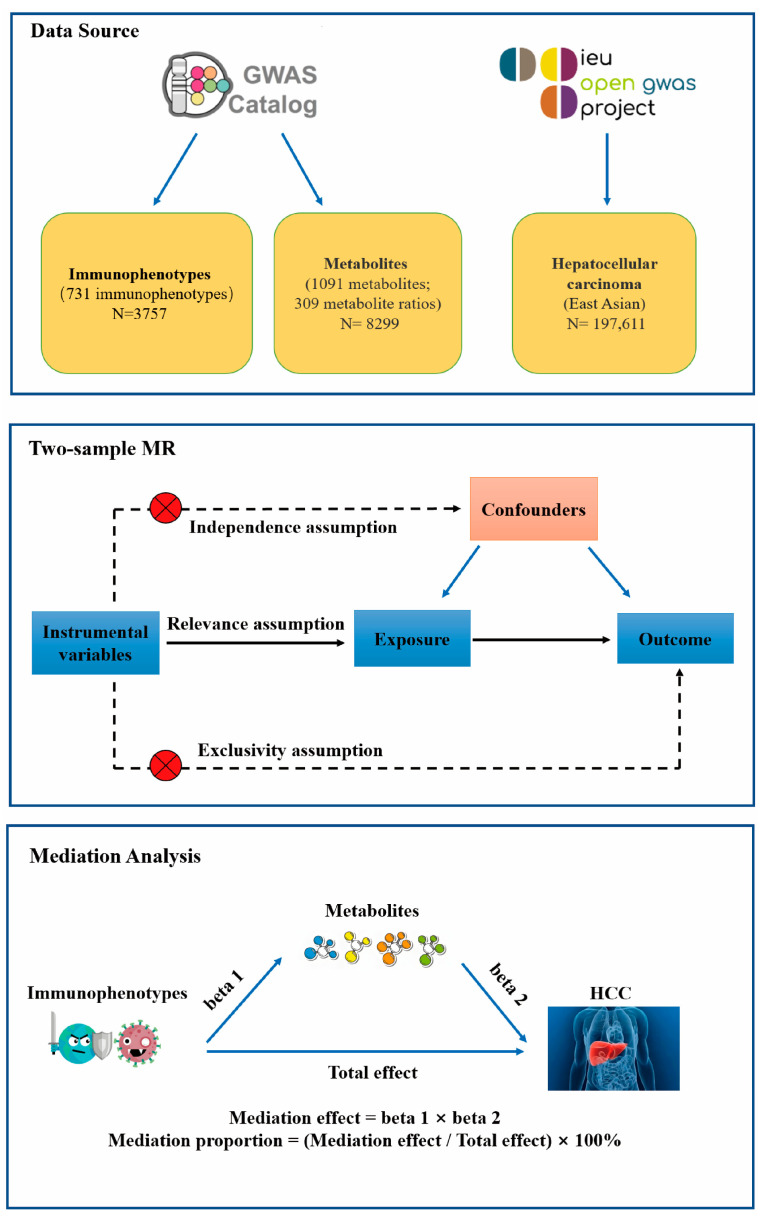
Flowchart of this research. HCC, hepatocellular carcinoma; MR, Mendelian randomization.

Figure 1 depicts the research flowchart.

### 3.1. The Overall Causal Impact of Immunocytes on HCC

**Figure 2:**
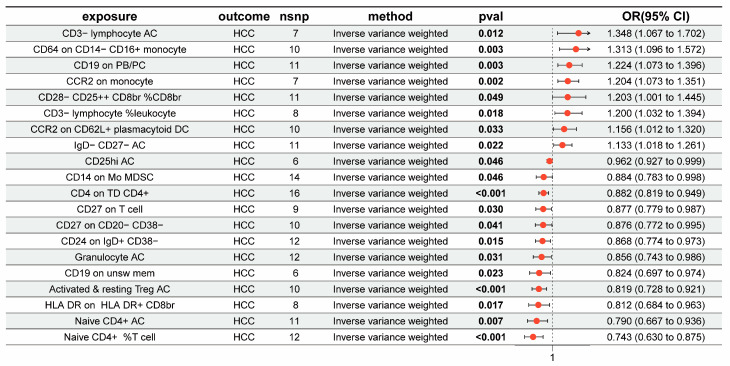
Forest plots depicting the causal impacts of immunocytes on HCC. OR, odds ratio; CI, confidence interval.

Figure 2 displays the outcomes of the IVW approach for seven groups of immunocytes in relation to HCC in East Asian populations. The results indicate a positive correlation between eight immunocytes and the progression of HCC (OR > 1, P_IVW_ < 0.05). cDC Panel: CCR2 on CD62L+ plasmacytoid DC and CCR2 on monocyte; Treg Panel: CD28− CD25++ CD8br %CD8br; B cell Panel: IgD− CD27− AC and CD19 on PB/PC; Monocyte Panel: CD64 on CD14− CD16+ monocyte; TBNK Panel: CD3− lymphocyte %leukocyte and CD3− lymphocyte AC. Conversely, 12 other traits were found to be inversely associated with HCC risk (OR < 1, P_IVW_ < 0.05). B cell Panel: CD19 on unsw mem, CD24 on IgD+ CD38−, CD27 on CD20− CD38−, and CD27 on T cell; Maturation stages of T cell Panel: Naive CD4+ %T cell, Naive CD4+ AC, and CD4 on TD CD4+; Myeloid cell Panel: CD14 on Mo MDSC; TBNK Panel: HLA DR on HLA DR+ CD8br and Granulocyte AC; Treg Panel: Activated and resting Treg AC and CD25hi AC. Afterward, we used an FDR adjustment on the outcomes of the IVW approach for immune cell characteristics. All 20 immunocytes that were analyzed before showed either strong or suggestive evidence of correlations with HCC (FDR < 0.2). The findings of the MR study are shown in Supplementary Table S1. Subsequently, we conducted a reverse MR study on the 20 immunocytes in connection with HCC, removing those exhibiting reverse causal impacts (Supplementary Figure S1). The MR–Egger regression examinations did not provide any evidence of horizontal pleiotropy. Nevertheless, Cochran’s Q statistics indicated a noteworthy variation in SNPs in CD14 on Mo MDSC (MR‒Egger: *p* = 0.0109; IVW: *p* = 0.0016) (Supplementary Table S2).

### 3.2. The Overall Causal Impact of Plasma Metabolites on HCC

**Figure 3:**
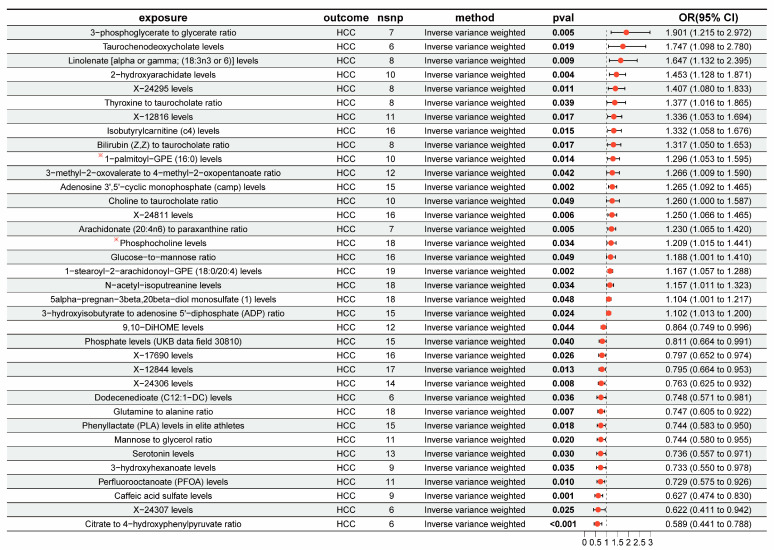
Forest plots depicting the causal impacts of plasma metabolites on HCC. ※ represents metabolites included in “glycerophospholipid metabolism”; OR, odds ratio; CI, confidence interval.

Thirty-six metabolites were linked to changes in the risk of HCC for East Asians (P_IVW_ < 0.05) (Figure 3). Among them, 21 metabolites increased the risk of HCC including the 3-phosphoglycerate-to-glycerate ratio (OR = 1.901, 95% CI [1.215–2.972], *p* = 0.005) and taurochenodeoxycholate levels (OR = 1.747, 95% CI [1.098–2.780], *p* = 0.019), while 15 metabolites were linked with the risk of HCC, such as the Citrate-to-4-hydroxyphenylpyruvate ratio (OR = 0.589, 95% CI [0.441–0.788], *p* < 0.01) and X-24307 levels (OR = 0.622, 95% CI [0.411–0.942], *p* = 0.025) (Supplementary Table S3). The sensitivity analyses provided evidence that there was no horizontal pleiotropy or heterogeneity in these results (Supplementary Table S4). Moreover, the analysis of metabolic pathways indicated that “glycerophospholipid metabolism”, which includes Phosphocholine and 1-palmitoyl-GPE (16:0), was importantly related to the progression of HCC (*p* = 0.0099) (Supplementary Table S5).

### 3.3. The Results of the Mediation Analysis

With the goal of understanding the fundamental processes involved in the genesis and progression of HCC, we conducted a mediation analysis to discover the causal chain from immunocytes to HCC, which is facilitated by plasma metabolites. This analysis specifically examined the immunocytes and plasma metabolites that were already linked to HCC in the prior MR study. Initially, the relationship between immunocytes and metabolites was assessed using a two-sample MR. Subsequently, 33 associations between immunocytes and metabolites were identified. The results of the five methods of the MR study are listed in Supplementary Table S6. These MR findings were corroborated by sensitivity analysis, with the exception of the MR–Egger intercept for N-acetyl-isoputreanine levels (*p* = 0.0230), which displayed horizontal pleiotropy and hence was removed from subsequent study (Supplementary Table S7). In conclusion, we identified 15 mediating interactions, of which 12 were supported by strong evidence and 3 showed promising evidence (Table 1). The mediation analysis indicated that Linolenate [α or γ; (18:3n3 or 6)] levels exhibited the highest mediation proportion at 19.3%, mediating CD19 on PB/PC to HCC, and also demonstrated significant positive mediation impacts (β =−0.0128, 95% CI [−0.0247, −0.0009], *p* = 0.0339) on CD14 on Mo MDSC and HCC, contributing a mediation proportion of 10.4%. In addition, 9,10-DiHOME levels exhibited strong negative mediation impacts (β = −0.0059, 95% CI [−0.0108, −0.0011], *p* = 0.0154) on CD4 on TD CD4+ and HCC with a 4.73% proportion. The pathway from CD4+ T cells to HCC was strongly mediated by X-24306 levels and X-24307 levels with a 6.71% proportion and a 14.9% proportion. Additionally, 3-hydroxyhexanoate levels showed significant positive mediation impacts (β = 0.0131, 95% CI [0.0024, 0.0238], *p* = 0.0166) on CCR2 on CD62L+ plasmacytoid DC and HCC, with a mediation proportion of 9.03%. The pathway from CR2 on CD62L+ plasmacytoid DC to HCC was also mediated by Dodecenedioate (C12:1-DC) levels with a 9.14% proportion. Concurrently, the mediation ratios from CD28− CD25++ CD8br %CD8br to HCC through 5alpha-pregnan-3beta,20beta-diol monosulfate (1) and N-acetyl-isoputreanine levels were 3.21% and 4.03%. The results of this study consistently demonstrated the effects of exposure factors, mediators, and outcome factors. This was supported by scatterplots and a leave-one-out analysis, which confirmed the reliable causal relationships in the two-sample MR study (Table 1; Supplementary Figures S2 and S3).

## 4. Discussion

Metabolic reprogramming and immune evasion are two pivotal hallmarks of cancer, facilitating tumor development and progression [36]. Research has demonstrated that changes in the metabolic activity of tumor cells are influenced to some extent by the enlistment of immune cells [37]. Moreover, an increasing number of studies have shown that abnormal metabolites or cancer metabolism intermediates are essential for controlling immune cell activation, differentiation, proliferation, and function [38,39]. Our study identified 15 mediating interactions that include 11 immune cells, 14 plasma metabolites, and one HCC trait in East Asian populations. The mediation analysis supported the hypothesis that plasma metabolites play a mediating role in the influence of immune cells on HCC pathogenesis. This finding has the potential to provide fresh insights into the mechanisms that drive the start and development of HCC, thereby uncovering novel targets for therapeutic intervention.

Our MR results revealed that Linolenate [α or γ; (18:3n3 or 6)] positively mediates the causal relationship between CD19 on PB/PC and HCC (ME = 0.0389, MP = 19.3%), while negatively mediating the causal relationship between CD14 on Mo MDSC and HCC (ME = −0.0128, MP = 10.4%). γ linolenic acid (GLA) and α-linolenic acid (ALA) are members of the ω-6 family of polyunsaturated fatty acids, which are converted into arachidonic acid via a series of elongation and desaturation reactions [40,41]. According to Cui et al., GLA exhibits a chemo-protective effect against DEN-induced HCC [42], and Feng et al. demonstrated that ALA influences HCC progression via the FXR/Wnt/β-catenin signaling pathway [43]. Additionally, one study [44] showed that Farnesoid X receptor (FXR) ablation in DCs enhanced Treg cell generation. Moreover, Chimaeric Antigen Receptors (CARs) are artificial receptors that modify the specificity and function of lymphocytes. CARs designed to target CD19 have shown exceptional effectiveness in treating B-cell cancers [45]. We observed a positive correlation between CD19 on PB/PC and HCC (OR = 1.224, 95% CI = 1.073–1.396). Tu et al. [46] reported that S100A9+CD14+ monocytes contribute to resistance to anti-PD-1 immunotherapy in advanced HCC by dampening T cell-mediated antitumor activity, while our findings imply that CD14 on Mo MDSC may reduce the risk of HCC. Collectively, these findings propose that Linolenate [α or γ; (18:3n3 or 6)] could serve as a mediator, potentially regulating the FXR/Wnt/β-catenin signaling pathway, thereby modulating the immunocyte effects on HCC.

CD4 on TD CD4+ cells is commonly acknowledged as CD4 on Terminally Differentiated CD4+ T cells, recognized as a biologically significant subset of T cells playing pivotal roles in combating viral infections, suppressing tumor growth, and regulating immune responses [47]. Research has indicated that CD4 T cell assistance is instrumental in sustaining and enhancing the functionality of effector CD8 T cells within tumor microenvironments, and recent studies have highlighted the pivotal role of CD4 T cell epitopes in augmenting CD8 T cell response in human cancers [48,49]. One study found a direct relationship between a robust and efficient CD4 T cell-mediated cytotoxic response, better survival rates, and lower rates of recurrence in HCC [50]. Our research aligns with these findings, suggesting that CD4 on TD CD4+ is negatively correlated with HCC risk. Further mediation analysis revealed that 9,10-DiHOME exerted a negative mediating effect (ME = −0.00596, MP = 4.73%), where 9,10-DiHOME is a byproduct of linolenic acid (LA) metabolism. LA is first transformed into 9,10-EpOME and then further transformed into 9,10-DiHOME by cytochrome P450 epoxygenases and epoxide hydrolase enzymes, respectively [51,52]. In a murine metabolite study, 9,10-DiHOME was found as lipophilic metabolites originating from the gut microbiota that possess Treg-inducing action [53]. These insights suggest that 9,10-DiHOME may act as a mediator, increasing Treg levels within the tumor microenvironment, thereby modulating CD4 on TD CD4+ effects on HCC. Furthermore, the pathway from CD4 on TD CD4+ to HCC is also mediated by the metabolites X-24306 and X-24307, accounting for 6.71% and 14.9% of the mediating effect, respectively.

We confirmed the presence of a cause-and-effect connection between 20 different immunocytes and HCC in East Asian populations. Our findings indicate a positive correlation between CCR2 on monocytes and the progression of HCC. This correlation supports the study by Li et al., who showed that blocking CCL2/CCR2 signaling through CCR2 knockout or the use of a CCR2 antagonist effectively suppressed malignant development and metastasis of HCC, decreased the likelihood of recurrence after surgery, and improved overall survival [54], which is consistent with our MR results. Studies have demonstrated that the compound 3-hydroxy anthranilic acid, which is derived from kynurenine, effectively inhibits the formation of tumors in the HCC model by decreasing the proportion of F4/80lo CD64 PD-L1+lo macrophages [55]. Furthermore, Tang et al. identified a potential increase in HCC risk associated with CD14 on CD14+ CD16− monocytes, aligning with our findings that CD64 on CD14− CD16+ monocytes is associated with an increased risk of HCC. Additionally, Shi et al. discovered that B lymphocytes infiltrating the tumor border exhibited a distinct memory phenotype (IgD−IgG+CD27−CD38−). These lymphocytes showed surface markers that are typically found on antigen-presenting cells and demonstrated the ability to kill HCC tumor cells [56], but our findings indicate that an increase in IgD-CD27-AC elevates the risk of HCC. Given that genetic variations are randomly distributed, MR analysis can offer more precise insights than experimental research. Previous outcomes might be triggered by some common confounders shared by HCC and IgD-CD27-AC. Jin et al. investigated the role of long intergenic non-protein coding RNA 942 in promoting HCC cell proliferation and converting naive CD4 T cells into inducible Treg (iTreg) cells, which are regulated by solute carrier family 7 member 11, suggesting a protective role for naive CD4+ T cells in HCC pathogenesis [57]. Moreover, the knockdown of Tubulin γ-1 (TUBG1) was shown to inhibit the proliferation, invasion, and migration of HCC cells, and TUBG1 expression was negatively correlated with CD4+ regulatory T cell [58], which is consistent with our MR findings and provides evidence for a causal link.

Metabolites are essential in the early detection of persons who are at significant risk and in preventing the development of HCC. Prior research on the metabolomics of individuals with liver cancer has demonstrated increased concentrations of metabolites (including aromatic amino acids and fatty acids) and reduced levels of metabolites (such as glutamine and secondary bile acid) in HCC patients [59,60,61,62]. Bu et al. demonstrated that palmitic acid from a high-fat diet can activate protein kinase B (PKB/AKT) and promote HCC [63]. Additionally, a recent study indicated that decreased propionyl-CoA metabolism facilitates metabolic reprogramming and promotes HCC [64]. The results of our study establish a cause-and-effect association between 36 plasma metabolites and HCC in East Asian populations. Caffeic acid could inhibit glucose-regulated protein 75 attenuating the anti-apoptosis abilities and multi-drug resistance of HCC and contribute to the inhibition of the growth and expansion of hepatocellular carcinoma cells through the regulation of the AKT/CyclinD1/p21/p27 pathways [65,66]. Thus, our MR results further corroborate the conclusions drawn from prior studies. Furthermore, dysregulated bile acids (BAs) are closely tied to liver diseases, and taurochenodeoxycholate (TCDCA) is a kind of hydrophobic BA that Yan et al. identified as a promoter of liver carcinogenesis [67]. Additionally, Perfluorooctanoic acid (PFOA) is a man-made chemical with a fluorinated alkyl chain. It stimulates the PI3K/AKT/mTOR/4E-BP1 signaling pathway, which promotes cancer growth in HCC cells [68]. However, our findings indicate that PFOA is associated with a reduced risk of HCC, suggesting that this discrepancy may stem from residual confounding rather than a substantiated causal relationship. Glycerophospholipids are the predominant phospholipids found in the cell membranes of mammals. They can be further classified into subcategories, including phosphatidylcholine (PC), phosphatidylethanolamine (PE), and phosphatidylserine [69]. Research has indicated that glycerophospholipid metabolism can be remodeled by exercise, lowering PC levels, and creating a microenvironment that is unfavorable to HCC [70]. Additionally, PCs can be used as possible indicators in the blood and assist in identifying patients with liver illness caused by the hepatitis B virus who are at risk of developing HCC [71]. Solute carrier family 27 member 4 (SLC27A4) is highly expressed in HCC. This overexpression of SLC27A4 promotes the uptake of mono-unsaturated fatty acids, which increases the levels of phosphatidylethanolamine (PE) in HCC cells. As a result, the cells become resistant to lipid peroxidation and ferroptosis, leading to their survival and progression [72].

This study was the first to use a holistic MR framework to analyze the causative links among immune cells, plasma metabolites, and HCC in East Asian populations. In addition, we utilize a two-step MR study and mediation analysis to establish a pathway connecting immunocytes to HCC mediated by plasma metabolites. MR offers distinct advantages over randomized controlled trials, including the mitigation of confounding factors and the circumvention of reverse causality biases. For the purpose of guaranteeing the reliability of our MR findings, we implemented a series of sensitivity analyses. Nonetheless, this study has specific constraints. The primary studies lack essential clinical data, such as age and sex, precluding more granular subgroup analyses for HCC. Second, the predominant inclusion of East Asian individuals in GWASs constrains the generalizability of our findings to other ethnic groups. In addition, our conclusions are currently based on theory and need to be further validated through additional empirical research conducted in experimental and clinical settings.

## 5. Conclusions

This study provides a comprehensive assessment of the causal links among immunocytes, plasma metabolites, and HCC. We identified 11 distinct immunocytes that are causally linked to HCC, which is mediated by 14 specific metabolites. Our study emphasizes the importance of the interaction among immunocytes, metabolites, and HCC. The results provide a new and valuable understanding of the mechanisms that cause and advance HCC, thus potentially improving treatment approaches for HCC.

## Figures and Tables

**Table 1:** Mediation effect of immune cells on HCC via plasma metabolites.

Exposure	Mediator	Outcome	Mediated Effect (95% Cl)	Exposure	*p*-Value
CCR2 on CD62L+ plasmacytoid DC	3-hydroxyhexanoate levels	HCC	0.0131 (0.0024, 0.0238)	9.03% (1.64%, 16.4%)	0.0166
CCR2 on CD62L+ plasmacytoid DC	Dodecenedioate (C12:1-DC) levels	HCC	0.0132 (0.0033, 0.0232)	9.14% (2.25%, 16%)	0.0093
CD3− lymphocyte %leukocyte	X-24811 levels	HCC	0.0128 (0.0018, 0.0239)	7.04% (0.96%, 13.1%)	0.0232
CD4 on TD CD4+	9,10-DiHOME levels	HCC	−0.00596 (−0.0108, −0.0011)	4.73% (8.56%, 0.90%)	0.0154
CD4 on TD CD4+	X-24306 levels	HCC	−0.00844 (−0.0169, −2.2× 10^−5^)	6.71% (13.4%, 0.02%)	0.0494
CD4 on TD CD4+	X-24307 levels	HCC	−0.0188 (−0.0335, −0.0040)	14.9% (26.6%, 3.19%)	0.0126
CD14 on Mo MDSC	Linolenate [α or γ; (18:3n3 or 6)] levels	HCC	−0.0128 (−0.0247, −0.0009)	10.4% (19.9%, 0.78%)	0.0339
CD19 on PB/PC	Linolenate [α or γ; (18:3n3 or 6)] levels	HCC	0.0389 (0.012, 0.0658)	19.3% (5.94%, 32.6%)	0.0046
CD19 on unsw mem	N-acetyl-isoputreanine levels	HCC	−0.00829 (−0.0163, −0.0002)	4.27% (8.42%, 0.13%)	0.0434
CD24 on IgD+ CD38-	3-methyl-2-oxovalerate to 4-methyl-2-oxopentanoate ratio	HCC	−0.00862 (−0.0162, −0.0010)	6.08% (11.4%, 0.72%)	0.0262
CD28− CD25++ CD8br %CD8br	5alpha-pregnan-3beta,20beta-diol monosulfate (1) levels	HCC	0.00593 (−0.0002, 0.0121)	3.21% (−0.10%, 6.52%)	0.0579
CD28− CD25++ CD8br %CD8br	N-acetyl-isoputreanine levels	HCC	0.00745 (−0.0001, 0.015)	4.03% (−0.07%, 8.13%)	0.0538
CD64 on CD14− CD16+ monocyte	Citrate to 4-hydroxyphenylpyruvate ratio	HCC	0.0327 (0.0045, 0.0607)	12% (1.69%, 22.3%)	0.0226
Granulocyte AC	Serotonin levels	HCC	−0.0155 (−0.0288, −0.0022)	9.96% (18.5%, 1.43%)	0.0221
HLA DR on HLA DR+ CD8br	Phenyllactate levels in elite athletes	HCC	−0.014 (−0.0281, 0.0001)	6.7% (13.5%, −0.07%)	0.0523

## Data Availability

This study investigated datasets that are accessible to the public including the GWAS Catalog database (https://www.ebi.ac.uk/gwas/), accessed on 1 July 2024; the IEU Open GWAS database (https://gwas.mrcieu.ac.uk/), accessed on 1 July 2024; and the LD link database (https://ldlink.nih.gov/?tab=ldtrait), accessed on 3 July 2024.

## Supplementary Materials

The following supporting information can be downloaded at https://www.mdpi.com/article/10.3390/genes15091183/s1, Figure S1. Reverse MR analysis of inverse variance weighting method results; Figure S2. Scatterplots for two-sample MR analysis of immune cells on HCC, immune cells on plasma metabolites, and plasma metabolites on HCC. The horizontal axis corresponds to the effect of SNPs on the exposure variable, whereas the vertical axis depicts the effect of SNPs on the outcome variable; Figure S3. Leave-one-out plots for two-sample MR analysis of immune cells on HCC, immune cells on plasma metabolites, and plasma metabolites on HCC. The dark dots represent the effect estimates from IVW MR analysis excluding the index SNPs. The red lines indicate the effect size derived from the pooled analysis including all SNPs according to the IVW MR method. Table S1. Information about five methods of Mendelian randomization (Immune cells and HCC); Table S2. Sensitive analysis of the association between identified immune cells and HCC; Table S3. Information about five methods of Mendelian randomization (Plasma metabolites and HCC); Table S4. Sensitive analysis of the association between identified plasma metabolites and HCC; Table S5. Metabolic pathway analysis of identified plasma metabolites; Table S6. Information about five methods of Mendelian randomization (Immune cells and Plasma metabolites); Table S7. Sensitive analysis of the association between identified immune cells on identified plasma metabolites.
