# Supplementary material for: Causal Effect of Immunocytes, Plasma Metabolites, and Hepatocellular Carcinoma: A Bidirectional Two-Sample Mendelian Randomization Study and Mediation Analysis in East Asian Populations
Source: Genes (Basel). 2024 Sep 9;15(9):1183. doi: 10.3390/genes15091183 (PMC11431556; doi:10.3390/genes15091183)
Supplement: Supplementary file 1 [file genes-15-01183-s001.zip › Supplementary Figure S1.pdf]

exposure	outcome	nsnp	method	pval	OR(95% CI)
HCC	Naive CD4+ AC	12	Inverse variance weighted	**0.036**	1.123 (1.008 to 1.252)
	CD3− lymphocyte AC	12	Inverse variance weighted	0.826	1.011 (0.914 to 1.120)
	CD25hi AC	12	Inverse variance weighted	0.124	1.107 (0.973 to 1.259)
	Granulocyte AC	12	Inverse variance weighted	0.727	1.018 (0.919 to 1.128)
	IgD− CD27− AC	12	Inverse variance weighted	0.629	1.025 (0.926 to 1.136)
	HLA DR on HLA DR+ CD8br	12	Inverse variance weighted	0.619	1.031 (0.914 to 1.163)
	CD14 on Mo MDSC	12	Inverse variance weighted	0.391	0.909 (0.732 to 1.130)
	CCR2 on CD62L+ plasmacytoid DC	12	Inverse variance weighted	0.987	1.001 (0.862 to 1.163)
	CD27 on T cell	12	Inverse variance weighted	0.484	0.954 (0.838 to 1.088)
	CD27 on CD20− CD38−	12	Inverse variance weighted	0.957	0.997 (0.877 to 1.132)
	CD24 on IgD+ CD38−	12	Inverse variance weighted	0.695	1.034 (0.875 to 1.221)
	CD28− CD25++ CD8br %CD8br	12	Inverse variance weighted	0.503	0.960 (0.853 to 1.081)
	CD19 on unsw mem	12	Inverse variance weighted	0.289	1.067 (0.947 to 1.202)
	CD3− lymphocyte %leukocyte	12	Inverse variance weighted	0.861	0.991 (0.894 to 1.098)
	CCR2 on monocyte	12	Inverse variance weighted	0.540	0.965 (0.863 to 1.080)
	CD4 on TD CD4+T cell	12	Inverse variance weighted	0.911	0.994 (0.890 to 1.109)
	Naive CD4+T cell	12	Inverse variance weighted	0.052	1.103 (0.999 to 1.218)
	CD19 on Plasma Blast−Plasma Cell	12	Inverse variance weighted	0.928	1.005 (0.904 to 1.117)
	CD64 on CD14− CD16+ monocyte	12	Inverse variance weighted	0.418	0.951 (0.841 to 1.075)
	Activated & resting CD4 regulatory T cell	12	Inverse variance weighted	0.697	1.020 (0.922 to 1.129)

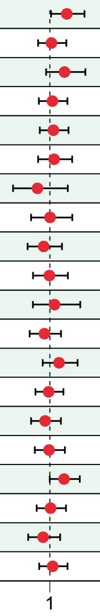

Supplementary Figure S1. Reverse MR analysis of inverse variance weighting method results
