# Supplementary material for: Causal Effect of Immunocytes, Plasma Metabolites, and Hepatocellular Carcinoma: A Bidirectional Two-Sample Mendelian Randomization Study and Mediation Analysis in East Asian Populations
Source: Genes (Basel). 2024 Sep 9;15(9):1183. doi: 10.3390/genes15091183 (PMC11431556; doi:10.3390/genes15091183)
Supplement: Supplementary file 1 [file genes-15-01183-s001.zip › Supplementary Figure S2.pdf]

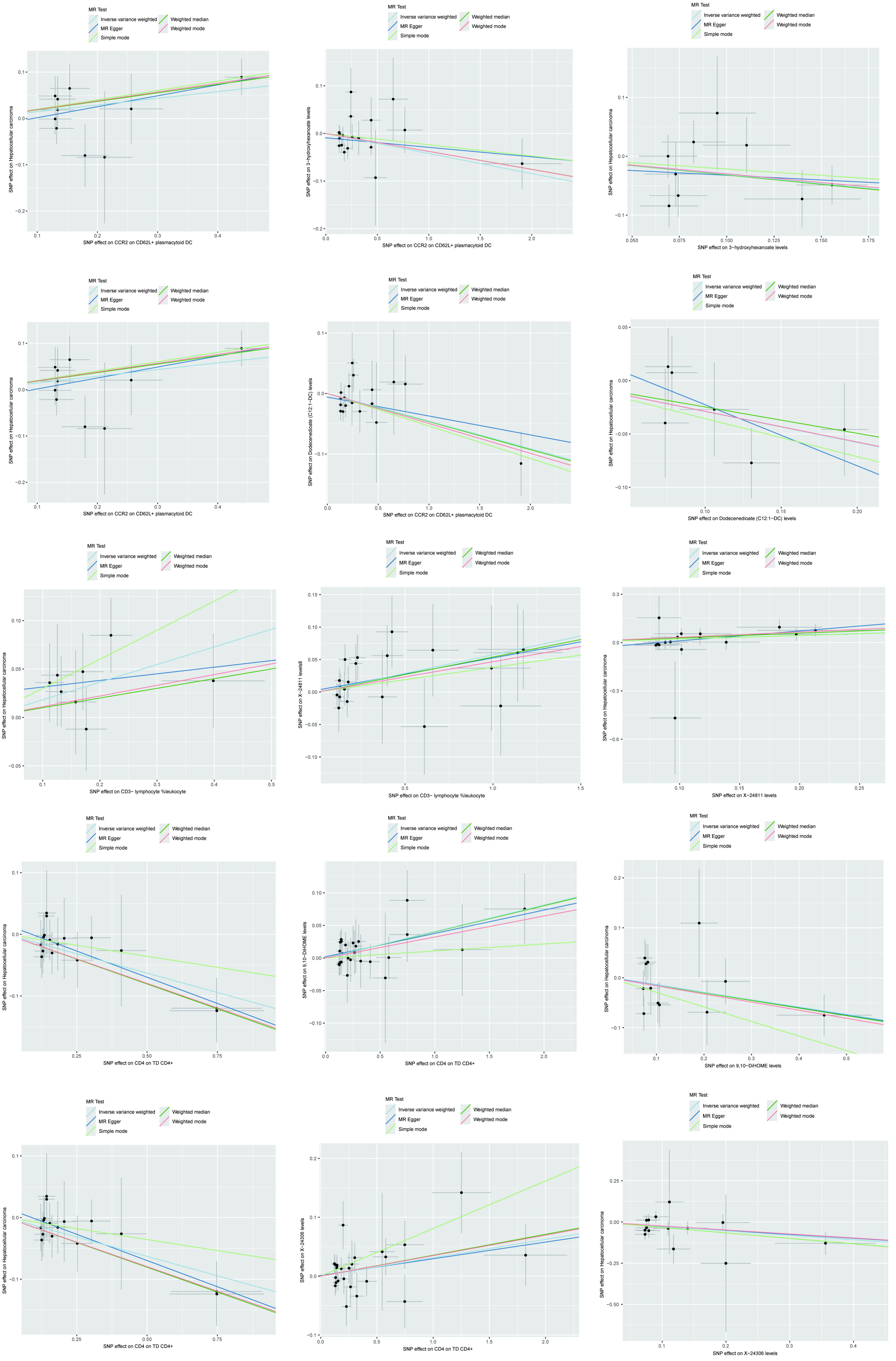
MR Test
Inverse variance weighted
MR Egger
Simple mode
Weighted median
Weighted mode
SNP effect on CCR2 on CD62L+ plasmacytoid DC
SNP effect on Hepatocellular carcinoma
SNP effect on 3−hydroxyhexanoate levels
SNP effect on Hepatocellular carcinoma
SNP effect on CCR2 on CD62L+ plasmacytoid DC
SNP effect on Dodecenedioate (C12:1−DC) levels
SNP effect on Dodecenedioate (C12:1−DC) levels
SNP effect on Hepatocellular carcinoma
SNP effect on CD3− lymphocyte %leukocyte
SNP effect on X−24811 levels
SNP effect on X−24811 levels
SNP effect on CD4 on TD CD4+
SNP effect on 9,10−DiHOME levels
SNP effect on 9,10−DiHOME levels
SNP effect on X−24306 levels
SNP effect on X−24306 levels

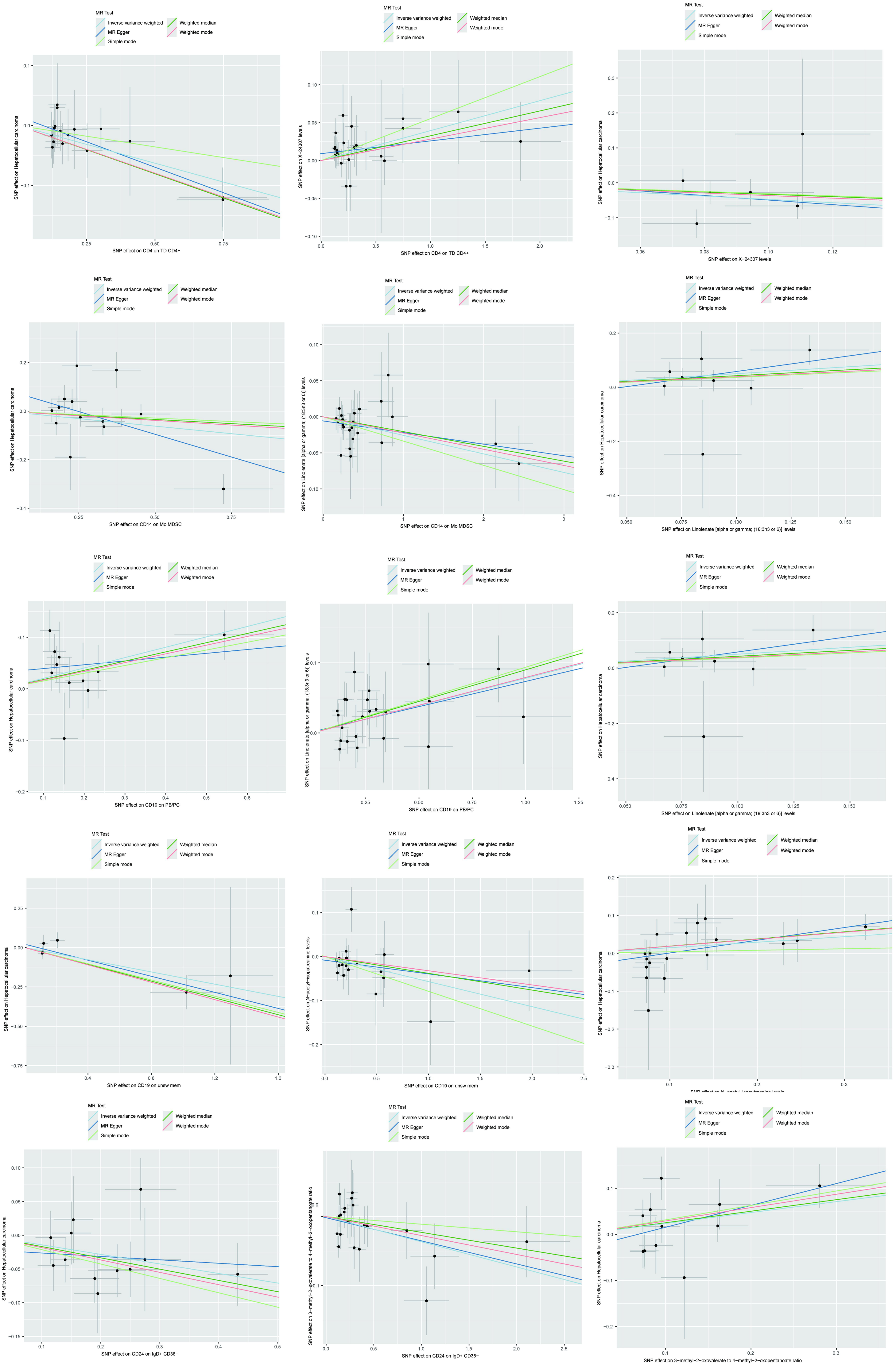
MR Test
Inverse variance weighted
MR Egger
Simple mode
Weighted median
Weighted mode
SNP effect on Hepatocellular carcinoma
SNP effect on CD4 on TD CD4+
SNP effect on X−24307 levels
SNP effect on CD14 on Mo MDSC
SNP effect on Linolenate [alpha or gamma; (18:3n3 or 6)] levels
SNP effect on CD19 on PB/PC
SNP effect on CD19 on unsw mem
SNP effect on N−acetyl−isoputreanine levels
SNP effect on CD24 on IgD+ CD38−
SNP effect on 3−methyl−2−oxovalerate to 4−methyl−2−oxopentanoate ratio

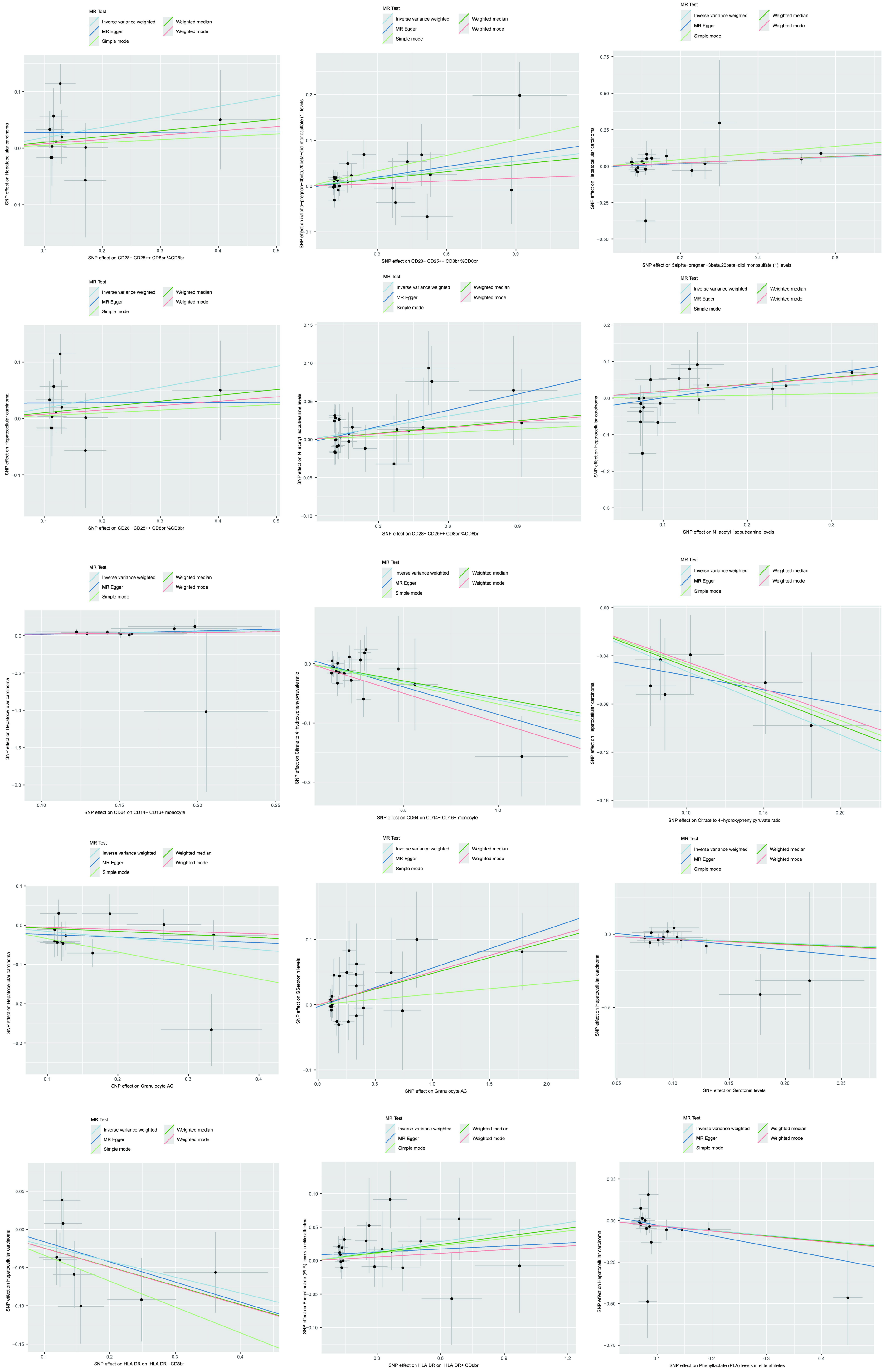

MR Test
Inverse variance weighted
MR Egger
Simple mode
Weighted median
Weighted mode
SNP effect on Hepatocellular carcinoma
SNP effect on CD28− CD25++ CD8br %CD8br
SNP effect on 5alpha−pregnan−3beta,20beta−diol monosulfate (1) levels
SNP effect on CD28− CD25++ CD8br %CD8br
SNP effect on Hepatocellular carcinoma
SNP effect on 5alpha−pregnan−3beta,20beta−diol monosulfate (1) levels
SNP effect on Hepatocellular carcinoma
SNP effect on CD28− CD25++ CD8br %CD8br
SNP effect on N−acetyl−isoputreanine levels
SNP effect on CD28− CD25++ CD8br %CD8br
SNP effect on Hepatocellular carcinoma
SNP effect on N−acetyl−isoputreanine levels
SNP effect on Hepatocellular carcinoma
SNP effect on CD64 on CD14− CD16+ monocyte
SNP effect on Citrate to 4−hydroxyphenylpyruvate ratio
SNP effect on CD64 on CD14− CD16+ monocyte
SNP effect on Hepatocellular carcinoma
SNP effect on Citrate to 4−hydroxyphenylpyruvate ratio
SNP effect on Hepatocellular carcinoma
SNP effect on Granulocyte AC
SNP effect on GSerotonin levels
SNP effect on Granulocyte AC
SNP effect on Hepatocellular carcinoma
SNP effect on Serotonin levels
SNP effect on Hepatocellular carcinoma
SNP effect on HLA DR on HLA DR+ CD8br
SNP effect on Phenyllactate (PLA) levels in elite athletes
SNP effect on HLA DR on HLA DR+ CD8br
SNP effect on Hepatocellular carcinoma
SNP effect on Phenyllactate (PLA) levels in elite athletes

Supplementary Figure S2. Scatterplots for two-sample MR analysis of immune cells on HCC, immune cells on plasma metabolites, and plasma metabolites on HCC. The horizontal axis corresponds to the effect of SNPs on the exposure variable, whereas the vertical axis depicts the effect of SNPs on the outcome variable.
