# Supplementary material for: Causal Effect of Immunocytes, Plasma Metabolites, and Hepatocellular Carcinoma: A Bidirectional Two-Sample Mendelian Randomization Study and Mediation Analysis in East Asian Populations
Source: Genes (Basel). 2024 Sep 9;15(9):1183. doi: 10.3390/genes15091183 (PMC11431556; doi:10.3390/genes15091183)
Supplement: Supplementary file 1 [file genes-15-01183-s001.zip › Supplementary Figure S3.pdf]

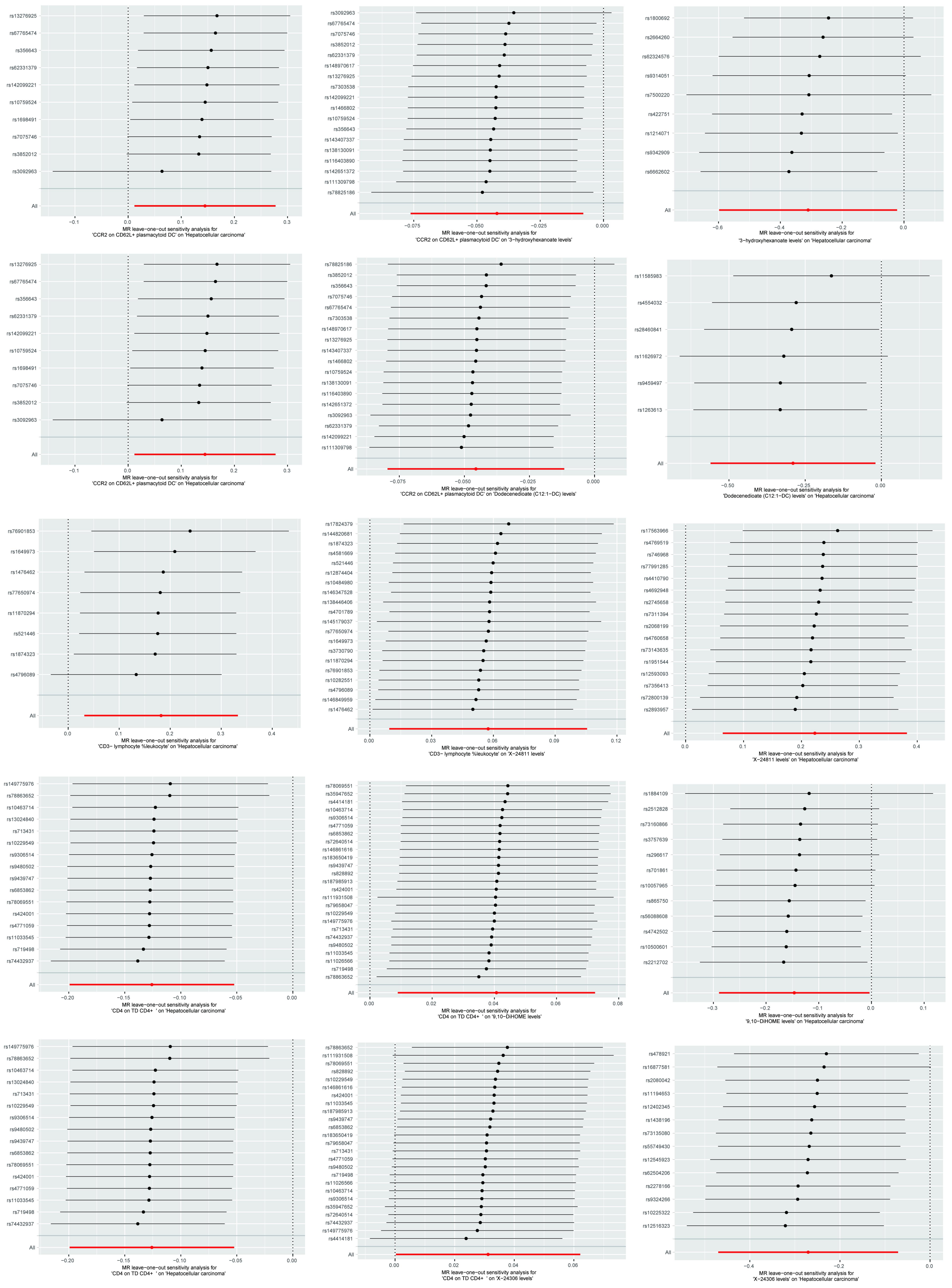
rs13276925
rs67765474
rs356643
rs62331379
rs142099221
rs10759524
rs1698491
rs7075746
rs3852012
rs3092963
All
MR leave−one−out sensitivity analysis for
'CCR2 on CD62L+ plasmacytoid DC' on 'Hepatocellular carcinoma'
rs3092963
rs67765474
rs7075746
rs3852012
rs62331379
rs148970617
rs13276925
rs7303538
rs142099221
rs1466802
rs10759524
rs356643
rs143407337
rs138130091
rs116403890
rs142651372
rs111309798
rs78825186
All
MR leave−one−out sensitivity analysis for
'CCR2 on CD62L+ plasmacytoid DC' on '3−hydroxyhexanoate levels'
rs1800692
rs2664260
rs62324576
rs9314051
rs7500220
rs422751
rs1214071
rs9342909
rs6662602
All
MR leave−one−out sensitivity analysis for
'3−hydroxyhexanoate levels' on 'Hepatocellular carcinoma'
rs13276925
rs67765474
rs356643
rs62331379
rs142099221
rs10759524
rs1698491
rs7075746
rs3852012
rs3092963
All
MR leave−one−out sensitivity analysis for
'CCR2 on CD62L+ plasmacytoid DC' on 'Hepatocellular carcinoma'
rs78825186
rs3852012
rs356643
rs7075746
rs67765474
rs7303538
rs148970617
rs13276925
rs143407337
rs1466802
rs10759524
rs138130091
rs116403890
rs142651372
rs3092963
rs62331379
rs142099221
rs111309798
All
MR leave−one−out sensitivity analysis for
'CCR2 on CD62L+ plasmacytoid DC' on 'Dodecenedioate (C12:1−DC) levels'
rs11585983
rs4554032
rs28460841
rs11626972
rs9459497
rs1263613
All
MR leave−one−out sensitivity analysis for
'Dodecenedioate (C12:1−DC) levels' on 'Hepatocellular carcinoma'
rs76901853
rs1649973
rs1476462
rs77650974
rs11870294
rs521446
rs1874323
rs4796089
All
MR leave−one−out sensitivity analysis for
'CD3− lymphocyte %leukocyte' on 'Hepatocellular carcinoma'
rs17824379
rs144820681
rs1874323
rs4581669
rs521446
rs12874404
rs10484980
rs146347528
rs138446406
rs4701789
rs145179037
rs77650974
rs1649973
rs3730790
rs11870294
rs76901853
rs10282551
rs4796089
rs146849959
rs1476462
All
MR leave−one−out sensitivity analysis for
'CD3− lymphocyte %leukocyte' on 'X−24811 levels'
rs17563966
rs4769519
rs746968
rs77991285
rs4410790
rs4692948
rs2745658
rs7311394
rs2068199
rs4760658
rs73143635
rs1951544
rs12593093
rs7356413
rs72800139
rs2893957
All
MR leave−one−out sensitivity analysis for
'X−24811 levels' on 'Hepatocellular carcinoma'
rs149775976
rs78863652
rs10463714
rs13024840
rs713431
rs10229549
rs9306514
rs9480502
rs9439747
rs6853862
rs78069551
rs424001
rs4771059
rs11033545
rs719498
rs74432937
All
MR leave−one−out sensitivity analysis for
'CD4 on TD CD4+ ' on 'Hepatocellular carcinoma'
rs78069551
rs35947652
rs4414181
rs10463714
rs9306514
rs4771059
rs6853862
rs72640514
rs146861616
rs183650419
rs9439747
rs828892
rs187985913
rs424001
rs111931508
rs79658047
rs10229549
rs149775976
rs713431
rs74432937
rs9480502
rs11033545
rs11026566
rs719498
rs78863652
All
MR leave−one−out sensitivity analysis for
'CD4 on TD CD4+ ' on '9,10−DiHOME levels'
rs1884109
rs2512828
rs73160866
rs3757639
rs296617
rs701861
rs10057965
rs865750
rs56088608
rs4742502
rs10500601
rs2212702
All
MR leave−one−out sensitivity analysis for
'9,10−DiHOME levels' on 'Hepatocellular carcinoma'
rs149775976
rs78863652
rs10463714
rs13024840
rs713431
rs10229549
rs9306514
rs9480502
rs9439747
rs6853862
rs78069551
rs424001
rs4771059
rs11033545
rs719498
rs74432937
All
MR leave−one−out sensitivity analysis for
'CD4 on TD CD4+ ' on 'Hepatocellular carcinoma'
rs78863652
rs111931508
rs78069551
rs828892
rs10229549
rs146861616
rs424001
rs11033545
rs187985913
rs9439747
rs6853862
rs183650419
rs79658047
rs713431
rs4771059
rs9480502
rs719498
rs11026566
rs10463714
rs9306514
rs35947652
rs72640514
rs74432937
rs149775976
rs4414181
All
MR leave−one−out sensitivity analysis for
'CD4 on TD CD4+ ' on 'X−24306 levels'
rs478921
rs16877581
rs2080042
rs11194653
rs12402345
rs1438196
rs73135080
rs55749430
rs12545923
rs62504206
rs2278166
rs9324266
rs10225322
rs12516323
All
MR leave−one−out sensitivity analysis for
'X−24306 levels' on 'Hepatocellular carcinoma'

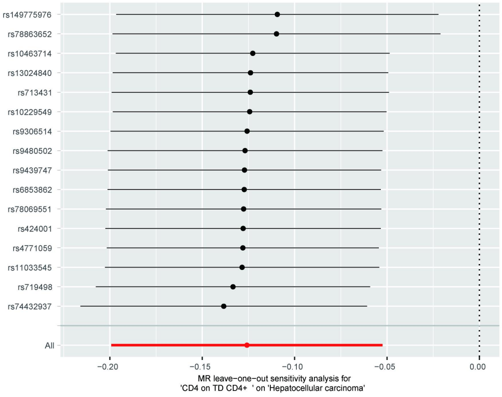
rs149775976
rs78863652
rs10463714
rs13024840
rs713431
rs10229549
rs9306514
rs9480502
rs9439747
rs6853862
rs78069551
rs424001
rs4771059
rs11033545
rs719498
rs74432937
All
−0.20
−0.15
−0.10
−0.05
0.00
MR leave−one−out sensitivity analysis for
'CD4 on TD CD4+ ' on 'Hepatocellular carcinoma'

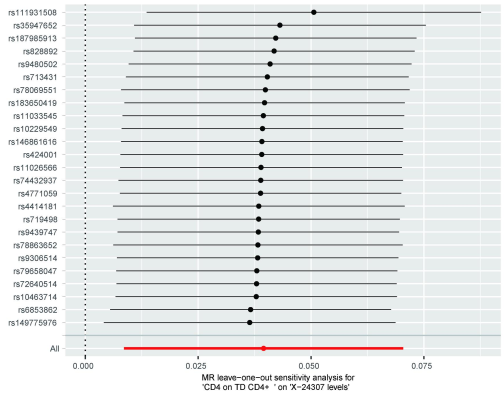
rs111931508
rs35947652
rs187985913
rs828892
rs9480502
rs713431
rs78069551
rs183650419
rs11033545
rs10229549
rs146861616
rs424001
rs11026566
rs74432937
rs4771059
rs4414181
rs719498
rs9439747
rs78863652
rs9306514
rs79658047
rs72640514
rs10463714
rs6853862
rs149775976
All
0.000
0.025
0.050
0.075
MR leave−one−out sensitivity analysis for
'CD4 on TD CD4+ ' on 'X−24307 levels'

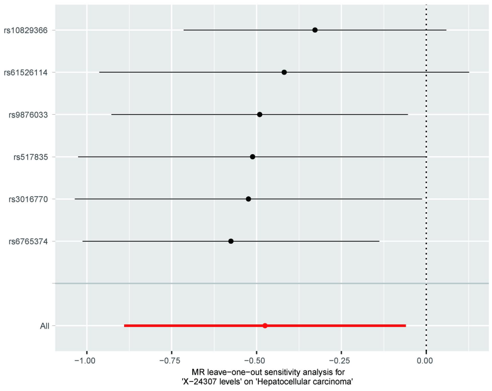
rs10829366
rs61526114
rs9876033
rs517835
rs3016770
rs6765374
All
−1.00
−0.75
−0.50
−0.25
0.00
MR leave−one−out sensitivity analysis for
'X−24307 levels' on 'Hepatocellular carcinoma'

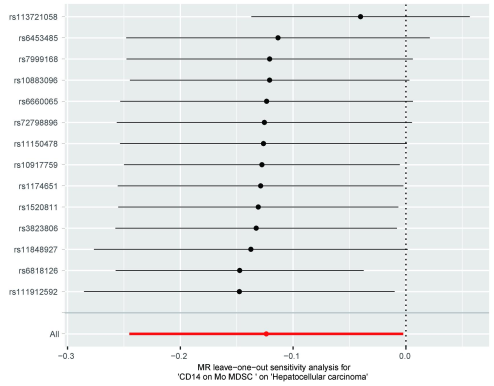
rs113721058
rs6453485
rs7999168
rs10883096
rs6660065
rs72798896
rs11150478
rs10917759
rs1174651
rs1520811
rs3823806
rs11848927
rs6818126
rs111912592
All
−0.3
−0.2
−0.1
0.0
MR leave−one−out sensitivity analysis for
'CD14 on Mo MDSC ' on 'Hepatocellular carcinoma'

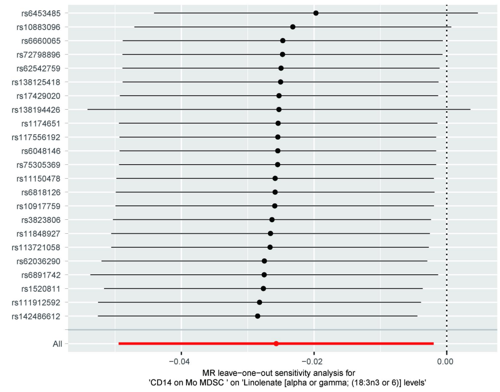
rs6453485
rs10883096
rs6660065
rs72798896
rs62542759
rs138125418
rs17429020
rs138194426
rs1174651
rs117556192
rs6048146
rs75305369
rs11150478
rs6818126
rs10917759
rs3823806
rs11848927
rs113721058
rs62036290
rs6891742
rs1520811
rs111912592
rs142486612
All
−0.04
−0.02
0.00
MR leave−one−out sensitivity analysis for
'CD14 on Mo MDSC ' on 'Linolenate [alpha or gamma; (18:3n3 or 6)] levels'

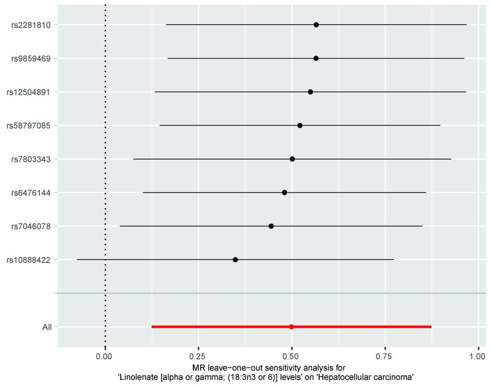
rs2281810
rs9859469
rs12504891
rs58797085
rs7803343
rs6476144
rs7046078
rs10888422
All
0.00
0.25
0.50
0.75
1.00
MR leave−one−out sensitivity analysis for
'Linolenate [alpha or gamma; (18:3n3 or 6)] levels' on 'Hepatocellular carcinoma'

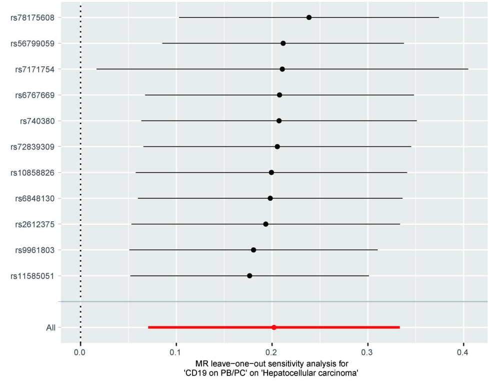
rs78175608
rs56799059
rs7171754
rs6767669
rs740380
rs72839309
rs10858826
rs6848130
rs2612375
rs9961803
rs11585051
All
0.0
0.1
0.2
0.3
0.4
MR leave−one−out sensitivity analysis for
'CD19 on PB/PC' on 'Hepatocellular carcinoma'

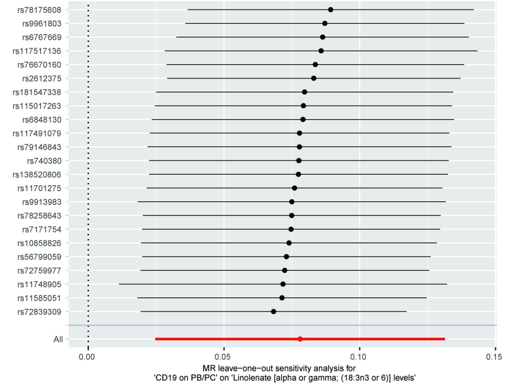
rs78175608
rs9961803
rs6767669
rs117517136
rs76670160
rs2612375
rs181547338
rs115017263
rs6848130
rs117491079
rs79146843
rs740380
rs138520806
rs11701275
rs9913983
rs78258643
rs7171754
rs10858826
rs56799059
rs72759977
rs11748905
rs11585051
rs72839309
All
0.00
0.05
0.10
0.15
MR leave−one−out sensitivity analysis for
'CD19 on PB/PC' on 'Linolenate [alpha or gamma; (18:3n3 or 6)] levels'

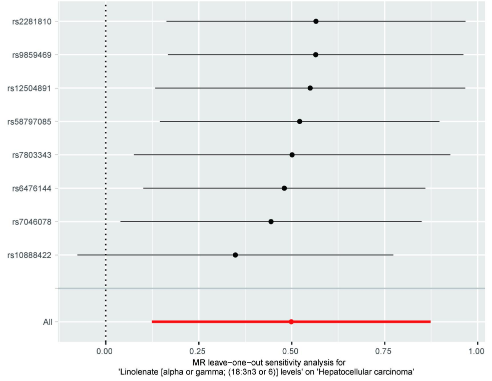
rs2281810
rs9859469
rs12504891
rs58797085
rs7803343
rs6476144
rs7046078
rs10888422
All
0.00
0.25
0.50
0.75
1.00
MR leave−one−out sensitivity analysis for
'Linolenate [alpha or gamma; (18:3n3 or 6)] levels' on 'Hepatocellular carcinoma'

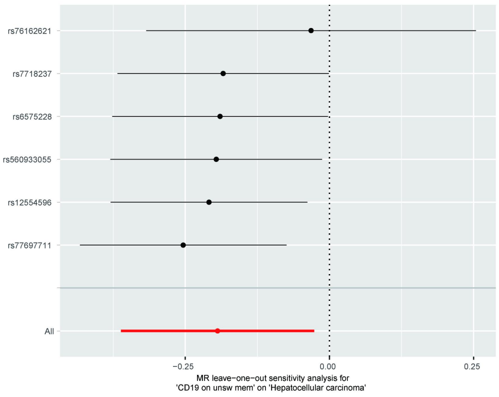
rs76162621
rs7718237
rs6575228
rs560933055
rs12554596
rs77697711
All
−0.25
0.00
0.25
MR leave−one−out sensitivity analysis for
'CD19 on unsw mem' on 'Hepatocellular carcinoma'

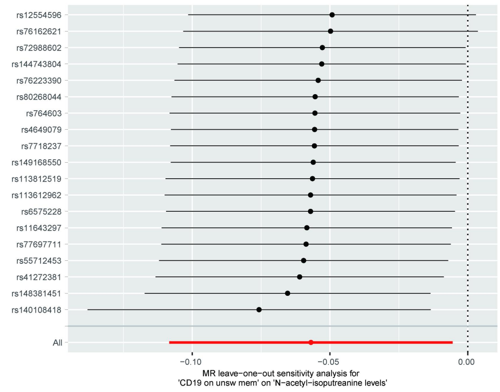
rs12554596
rs76162621
rs72988602
rs144743804
rs76223390
rs80268044
rs764603
rs4649079
rs7718237
rs149168550
rs113812519
rs113612962
rs6575228
rs11643297
rs77697711
rs55712453
rs41272381
rs148381451
rs140108418
All
−0.10
−0.05
0.00
MR leave−one−out sensitivity analysis for
'CD19 on unsw mem' on 'N−acetyl−isoputreanine levels'

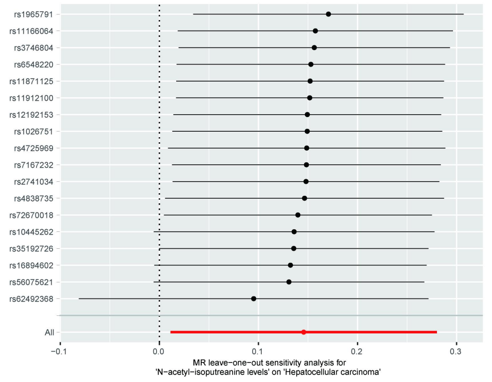
rs1965791
rs11166064
rs3746804
rs6548220
rs11871125
rs11912100
rs12192153
rs1026751
rs4725969
rs7167232
rs2741034
rs4838735
rs72670018
rs10445262
rs35192726
rs16894602
rs56075621
rs62492368
All
−0.1
0.0
0.1
0.2
0.3
MR leave−one−out sensitivity analysis for
'N−acetyl−isoputreanine levels' on 'Hepatocellular carcinoma'

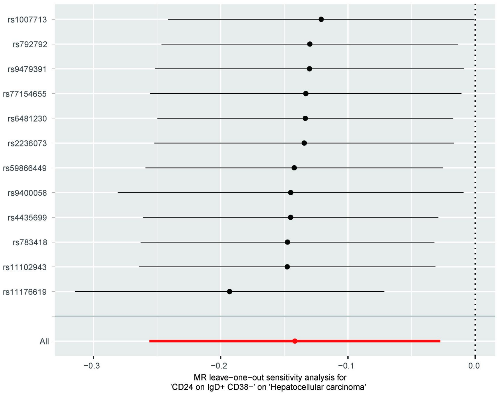
rs1007713
rs792792
rs9479391
rs77154655
rs6481230
rs2236073
rs59866449
rs9400058
rs4435699
rs783418
rs11102943
rs11176619
All
−0.3
−0.2
−0.1
0.0
MR leave−one−out sensitivity analysis for
'CD24 on IgD+ CD38−' on 'Hepatocellular carcinoma'

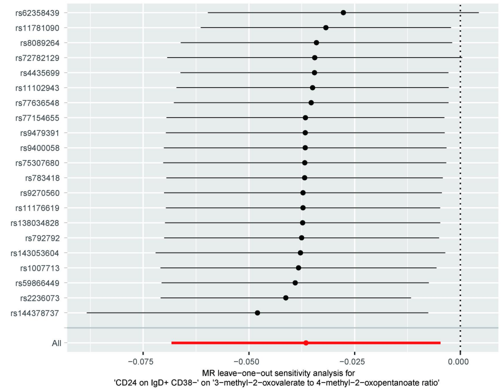
rs62358439
rs11781090
rs8089264
rs72782129
rs4435699
rs11102943
rs77636548
rs77154655
rs9479391
rs9400058
rs75307680
rs783418
rs9270560
rs11176619
rs138034828
rs792792
rs143053604
rs1007713
rs59866449
rs2236073
rs144378737
All
−0.075
−0.050
−0.025
0.000
MR leave−one−out sensitivity analysis for
'CD24 on IgD+ CD38−' on '3−methyl−2−oxovalerate to 4−methyl−2−oxopentanoate ratio'

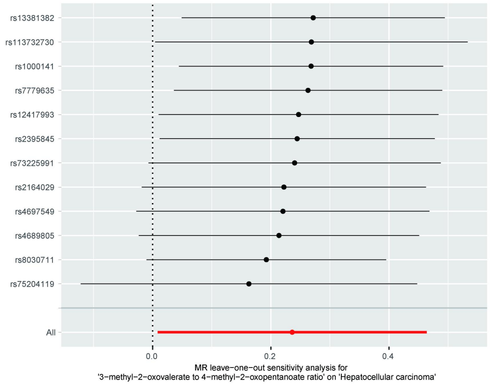
rs13381382
rs113732730
rs1000141
rs7779635
rs12417993
rs2395845
rs73225991
rs2164029
rs4697549
rs4689805
rs8030711
rs75204119
All
0.0
0.2
0.4
MR leave−one−out sensitivity analysis for
'3−methyl−2−oxovalerate to 4−methyl−2−oxopentanoate ratio' on 'Hepatocellular carcinoma'

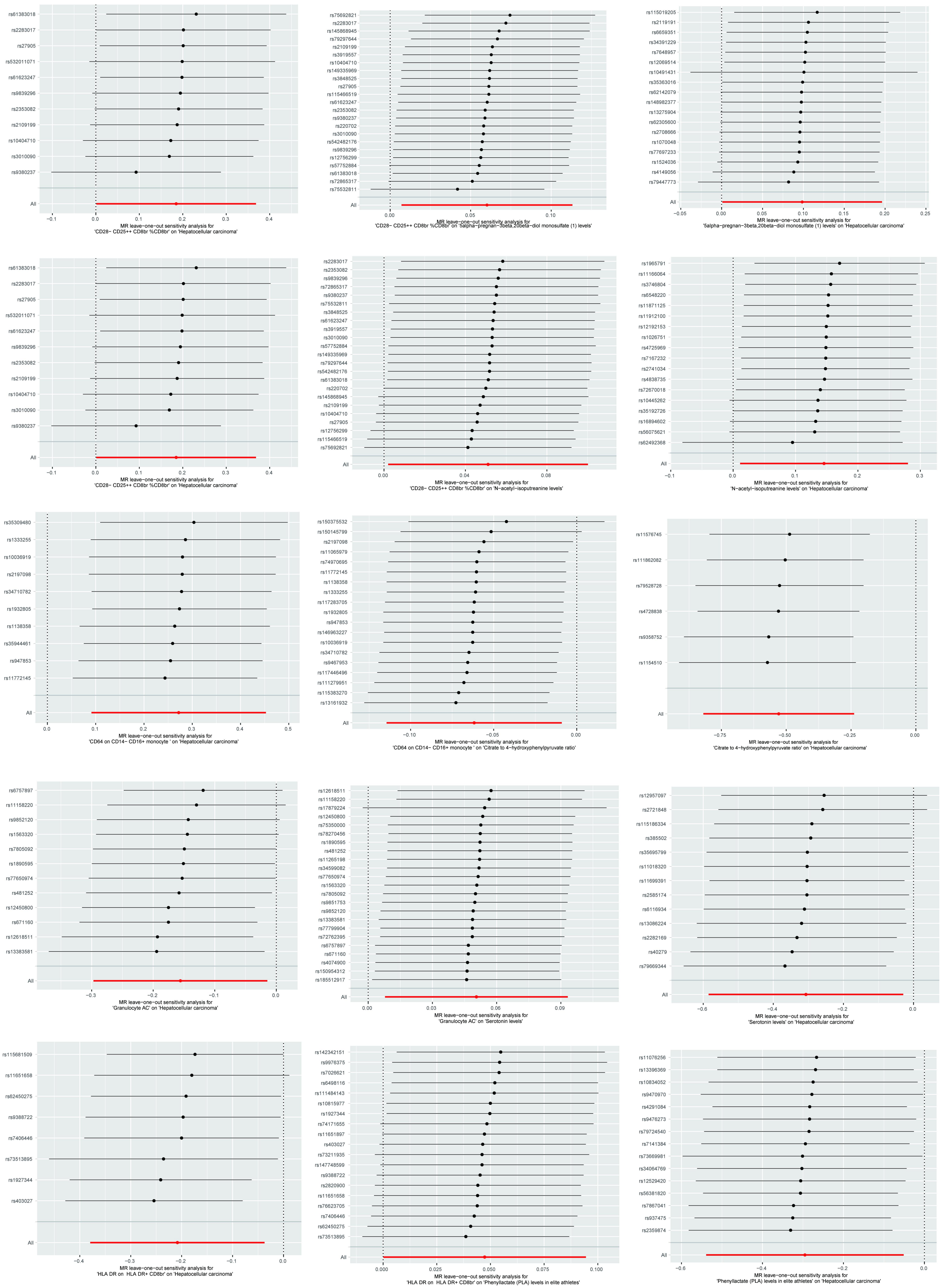

rs61383018
rs2283017
rs27905
rs532011071
rs61623247
rs9839296
rs2353082
rs2109199
rs10404710
rs3010090
rs9380237
All
MR leave−one−out sensitivity analysis for
'CD28− CD25++ CD8br %CD8br' on 'Hepatocellular carcinoma'
rs75692821
rs2283017
rs145868945
rs79297644
rs2109199
rs3919557
rs10404710
rs149335969
rs3848525
rs27905
rs115466519
rs61623247
rs2353082
rs9380237
rs220702
rs3010090
rs542482176
rs9839296
rs12756299
rs57752884
rs61383018
rs72865317
rs75532811
All
MR leave−one−out sensitivity analysis for
'CD28− CD25++ CD8br %CD8br' on '5alpha−pregnan−3beta,20beta−diol monosulfate (1) levels'
rs115019205
rs2119191
rs6659351
rs34391229
rs7648957
rs12069514
rs10491431
rs35363016
rs62142079
rs148982377
rs13275904
rs62305600
rs2708666
rs1070048
rs77697233
rs1524036
rs4149056
rs79447773
All
MR leave−one−out sensitivity analysis for
'5alpha−pregnan−3beta,20beta−diol monosulfate (1) levels' on 'Hepatocellular carcinoma'
rs61383018
rs2283017
rs27905
rs532011071
rs61623247
rs9839296
rs2353082
rs2109199
rs10404710
rs3010090
rs9380237
All
MR leave−one−out sensitivity analysis for
'CD28− CD25++ CD8br %CD8br' on 'Hepatocellular carcinoma'
rs2283017
rs2353082
rs9839296
rs72865317
rs9380237
rs75532811
rs3848525
rs61623247
rs3919557
rs3010090
rs57752884
rs149335969
rs79297644
rs542482176
rs61383018
rs220702
rs145868945
rs2109199
rs10404710
rs27905
rs12756299
rs115466519
rs75692821
All
MR leave−one−out sensitivity analysis for
'CD28− CD25++ CD8br %CD8br' on 'N−acetyl−isoputreanine levels'
rs1965791
rs11166064
rs3746804
rs6548220
rs11871125
rs11912100
rs12192153
rs1026751
rs4725969
rs7167232
rs2741034
rs4838735
rs72670018
rs10445262
rs35192726
rs16894602
rs56075621
rs62492368
All
MR leave−one−out sensitivity analysis for
'N−acetyl−isoputreanine levels' on 'Hepatocellular carcinoma'
rs35309480
rs1333255
rs10036919
rs2197098
rs34710782
rs1932805
rs1138358
rs35944461
rs947853
rs11772145
All
MR leave−one−out sensitivity analysis for
'CD64 on CD14− CD16+ monocyte ' on 'Hepatocellular carcinoma'
rs150375532
rs150145799
rs2197098
rs11065979
rs74970695
rs11772145
rs1138358
rs1333255
rs117283705
rs1932805
rs947853
rs146963227
rs10036919
rs34710782
rs9467953
rs117446496
rs111279951
rs115383270
rs13161932
All
MR leave−one−out sensitivity analysis for
'CD64 on CD14− CD16+ monocyte ' on 'Citrate to 4−hydroxyphenylpyruvate ratio'
rs11576745
rs111862082
rs79528728
rs4728838
rs9358752
rs1154510
All
MR leave−one−out sensitivity analysis for
'Citrate to 4−hydroxyphenylpyruvate ratio' on 'Hepatocellular carcinoma'
rs6757897
rs11158220
rs9852120
rs1563320
rs7805092
rs1890595
rs77650974
rs481252
rs12450800
rs671160
rs12618511
rs13383581
All
MR leave−one−out sensitivity analysis for
'Granulocyte AC' on 'Hepatocellular carcinoma'
rs12618511
rs11158220
rs17879224
rs12450800
rs75350000
rs78270456
rs1890595
rs481252
rs11265198
rs34599082
rs77650974
rs1563320
rs7805092
rs9851753
rs9852120
rs13383581
rs77799904
rs72762395
rs6757897
rs671160
rs4074900
rs150954312
rs185512917
All
MR leave−one−out sensitivity analysis for
'Granulocyte AC' on 'Serotonin levels'
rs12957097
rs2721848
rs115186334
rs385502
rs35695799
rs11018320
rs11699391
rs2585174
rs6116934
rs13086224
rs2282169
rs40279
rs79669344
All
MR leave−one−out sensitivity analysis for
'Serotonin levels' on 'Hepatocellular carcinoma'
rs115681509
rs11651658
rs62450275
rs9388722
rs7406446
rs73513895
rs1927344
rs403027
All
MR leave−one−out sensitivity analysis for
'HLA DR on HLA DR+ CD8br' on 'Hepatocellular carcinoma'
rs142342151
rs9976375
rs7026621
rs6498116
rs111484143
rs10815977
rs1927344
rs74171655
rs11651897
rs403027
rs73211935
rs147748599
rs9388722
rs2820900
rs11651658
rs76623705
rs7406446
rs62450275
rs73513895
All
MR leave−one−out sensitivity analysis for
'HLA DR on HLA DR+ CD8br' on 'Phenyllactate (PLA) levels in elite athletes'
rs11076256
rs13396369
rs10834052
rs9470970
rs4291084
rs9476273
rs79724540
rs7141384
rs73669981
rs34064769
rs12529420
rs56381820
rs7867041
rs937475
rs2359874
All
MR leave−one−out sensitivity analysis for
'Phenyllactate (PLA) levels in elite athletes' on 'Hepatocellular carcinoma'

Supplementary Figure S3. Leave-one-out plots for two-sample MR analysis of immune cells on HCC, immune cells on plasma metabolites, and plasma metabolites on HCC. The dark dots represent the effect estimates from IVW MR analysis excluding the index SNPs. The red lines indicate the effect size derived from the pooled analysis including all SNPs according to the IVW MR method.
